# Expectations for dog ownership: Perceived physical, mental and psychosocial health consequences among prospective adopters

**DOI:** 10.1371/journal.pone.0200276

**Published:** 2018-07-06

**Authors:** Lauren Powell, Debbie Chia, Paul McGreevy, Anthony L. Podberscek, Kate M. Edwards, Brendon Neilly, Adam J. Guastella, Vanessa Lee, Emmanuel Stamatakis

**Affiliations:** 1 Charles Perkins Centre, Prevention Research Collaboration, Sydney School of Public Health, Faculty of Medicine and Health, University of Sydney, Sydney, New South Wales, Australia; 2 Sydney School of Veterinary Science, University of Sydney, New South Wales, Australia; 3 Charles Perkins Centre, Sydney School of Veterinary Science, University of Sydney, New South Wales, Australia; 4 Charles Perkins Centre, Faculty of Health Sciences, University of Sydney, Sydney, New South Wales, Australia; 5 Royal Society for the Prevention of Cruelty to Animals (RSPCA) NSW, Sydney, New South Wales, Australia; 6 Autism Clinic for Translational Research, Brain and Mind Centre, Central Clinical School, Sydney Medical School, University of Sydney, Camperdown, New South Wales, Australia; Sapir College, ISRAEL

## Abstract

Dog ownership is popular worldwide, with most human-dog dyads forming successful attachment bonds. However, millions of dogs are surrendered to animal shelters annually, possibly due to mismatches between owner expectations and the realities of dog ownership. The aim of the current study was to explore the benefits and challenges people expect from dog ownership and how these expectations vary with previous ownership history. An Australian-wide sample of 3465 prospective adopters completed a self-administered online questionnaire about the physical, mental and psychosocial health benefits and challenges they associated with dog ownership. Among the potential benefits, respondents expected increased walking (89%), happiness (89%) and companionship (61%) and decreased stress (74%) and loneliness (61%). Among the challenges, they expected increased responsibility (64%) and dog training (62%). Ownership history influenced respondents’ expectations, with previous/current dog owners having consistently greater odds of expecting benefits and reduced odds of expecting challenges than non-owners. A possible explanation is that previous/current dog owners’ exhibit bias when considering dog ownership by selectively recalling positive experiences from previous ownership. Our findings support the need for education of prospective dog owners to ensure their expectations align with the reality of ownership, based on current scientific evidence.

## Introduction

Dog ownership is common around the world with 39% of Australian, 50% of USA and 27% of UK households owning a dog [1]. The vast majority of owners report a high level of satisfaction with their dog [2]. Nevertheless, millions of companion animals are relinquished to animal shelters each year [3]. In the USA, it is estimated that 3.3 million stray or surrendered dogs (or 10.2 dogs/1000 residents) are admitted to animal shelters annually. A large proportion of these dogs (20.3%) are euthanized [4]. Similar annual statistics are seen in Australia, with 9.3 dogs/1000 residents being admitted to welfare organisations and 43,900 dogs (20% of all admissions) being euthanized [5].

Relinquishment to animal shelters has a negative effect on the health of both owner [6] and dog [7]. Shelter environments are known to increase canine stress due to restrictions in space and social isolation [8]. The surrender process also places a strain on the mental wellbeing of some relinquishing owners, with many procrastinating and increasing their tolerance of dog-related problems prior to relinquishment [6].

The surrender of an animal can occur for a variety of reasons including behavioural problems, owner ill-health, lack of time and unanticipated costs [2, 9–13]. A disparity between owner expectations and the reality of companion animal ownership is also known to influence the risk of relinquishment [14]. For example, the presence of problematic behaviours is regularly cited as a primary reason for relinquishment [10, 11, 15]. However, the ability of owners to correctly identify canine behaviour is poor [16], despite most dog owners rating their understanding of dog behaviour highly [17]. Therefore, relinquishment is likely to at least partially reflect the owner’s perception that a behaviour is a problem, due to unmet expectations [18] or a disparity between owner lifestyle and the needs of the dog, rather than simply canine behavioural disorders.

The human-dog relationship is influenced by both canine and human characteristics [19], with previous research suggesting owner factors can affect canine behaviour and affective state [20]. Younger [21] or low socioeconomic status dog owners [22] have a greater risk of reporting canine behavioural problems. Previous ownership experience also plays a role, with first-time dog owners reporting a higher prevalence of problem behaviours such as fear, over-excitability [23, 24] and owner-directed aggression [23, 25].

In successful human-dog dyads, dog ownership is often associated with physical, mental and psychosocial health benefits, although the literature is incongruent. Most studies have shown dog owners have greater levels of physical activity than non-owners [26–30]. Some research suggests dog ownership is associated with improved cardiovascular functioning [31] and lower risk of cardiovascular and all-cause mortality [32], whilst other studies show no association between dog ownership and all-cause mortality [33, 34]. It has been proposed that dogs influence mental wellbeing by moderating the human physiological response to stress, specifically via attenuation of the hypothalamic-pituitary-adrenal (HPA) axis and heart rate response to stressors [35–37]. The ability of dogs to act as catalysts for social interactions is also thought to benefit human psychosocial health [38].

Despite substantial interest in the influence of dog ownership on human health outcomes, research investigating the expectations of prospective dog owners for benefits and challenges of ownership is scarce. Early research suggests the primary expectation and reason for acquisition of a companion animal is for companionship [23, 24, 39]. A more recent pilot study of 7 prospective dog adopters found undesirable animal behaviour was the most commonly expected challenge [40]. An additional study of 85 prospective dog adopters found owner factors such as relationship status and animal-care knowledge can explain some of the variation seen in expectations for dog ownership [41].

The expectations of prospective owners are likely to play a significant role in their decision to acquire a dog, and their overall satisfaction with the dog, thus influencing the likelihood of a positive human-dog relationship. Consequently, the aims of this study were to use an Australian-wide sample of prospective dog owners to *a)* explore expectations of the physical, mental and psychosocial health benefits and challenges from dog ownership and *b)* investigate how these expectations differ with dog ownership history and current status.

## Methods

An anonymous online survey was conducted from May 4^th^ to October 25^th^ 2016 among a cohort of potential dog owners. Participants were recruited through convenience sampling; potential prospective dog adopters who visited Pet Rescue’s website (www.petrescue.com.au), and clicked on ‘search for a dog’ were presented with a pop-up screen asking if they were willing to take part in an online survey. Pet Rescue is an Australia-wide animal welfare charity with an online directory of homeless pets and pet adoption organisations [42]. After providing informed consent, respondents over the age of 18 years were invited to complete the survey. Ethical approval was obtained from the Human Research Ethics Committee of the University of Sydney (2015/746).

Respondents were eligible to be included in the analyses if they completed at least 70% or more of the survey and reported a plausible age value (set to <103 years, which was the age of the oldest participant in a large epidemiological New South Wales cohort study [43]. The 70% completion criterion was set to ensure the analytical sample was relatively consistent across outcomes.

### Questionnaire

Data were retrieved from specific questions that formed part of a larger questionnaire. Questions relating to the current analysis explored reasons for considering dog adoption; perceived physical, mental, and psychosocial health benefits from owning a dog, and possible challenges of dog ownership (S1 Table). Further questions (reported elsewhere) asked about participants’ interests in and preferences for incentives to participate in future research studies about dog ownership; and whether they would consider delaying adoption of a dog by up to 3 months for the purpose of research [44]. Information on participants’ demographic characteristics, including age, gender, education level, and dog ownership status (never owned a dog, previous dog owner, current dog owner) was also obtained. Previous dog ownership status refers to whether the participant had previously owned a dog within the last 10 years.

### Data analysis

Multiple logistic regression models were used to examine the associations between dog ownership status and the four most common stated mental, physical, and psychosocial health benefits thought to be associated with dog ownership. The same analytical methods were used to describe the six most common expected challenges of dog ownership. Only the four/six most common reasons for each category are reported in the current analysis due to manuscript and analyses limitations; all remaining reasons were excluded. The referent category of dog ownership status for each analysis was ‘never owned a dog’. Model 1 was unadjusted while Model 2 was adjusted for age, sex, and education level. Statistical significance was defined as a two-tailed p ≤ 0.05. All statistical analyses were performed with SPSS software (version 22.0; SPSS Inc, Chicago, IL).

## Results

The full dataset included 3,919 participants. Of these, 449 participants were excluded for completing less than 70% of survey questions or not providing details of their age and gender. Seven participants were excluded due to implausible age values and another seven were excluded due to missing responses to questions. The final analysis included a sample of 3,465 participants. Descriptive statistics of the sample are presented in Table 1. The majority of participants were female (85%), younger (18–44 years; 52%), and university educated (56%). Over half (55%) of participants had previously owned a dog, 39% currently owned a dog, and 6% have never owned a dog.

**Table 1:** Characteristics of total survey sample and expectations for benefits and challenges by previous dog ownership status.

Variable	N (total n = 3465)	Never owned a dog (%)	Previous dog owner (%)	Current dog owner (%)	Total sample (%)
**Gender**					
Male	528	16.0	18.0	12.0	15.0
Female	2937	85.0	82.0	88.0	85.0
**Age (Years)**					
18–44	1812	78.0	51.0	50.0	52.0
45–64	1350	22.0	38.0	43.0	39.0
65+	303	1.0	10.0	8.0	9.0
**Education**					
Less than year 12 or equivalent	276	5.0	8.0	8.0	8.0
Year 12 or equivalent	639	20.0	17.0	20.0	19.0
Trade Certificate/Diploma	614	14.0	19.0	17.0	18.0
Bachelor’s Degree/Postgrad	1910	62.0	55.0	55.0	56.0
**Physical health benefits**					
Increase in walking	3094	87.0	91.5	86.5	89.3
Increase in physical fitness	1785	52.0	53.9	48.1	51.5
Weight loss	1030	31.5	32.6	25.5	29.7
Decrease in blood pressure	1374	30.0	38.6	42.6	39.7
**Mental health benefits**					
Increase in happiness	3097	85.0	88.5	91.2	89.4
Decreased stress	2575	67.0	73.6	76.4	74.3
Reduced depression	1966	52.5	54.4	60.6	56.7
Decreased loneliness	2103	55.0	58.7	64.3	60.7
**Social wellbeing benefits**					
Meet new friends	1763	49.0	50.9	51.1	50.9
Meet new partner	251	12.5	6.9	7.0	7.2
Get to know neighbourhood	1302	33.0	37.5	38.3	37.6
Companionship from dog	2117	56.0	61.5	61.3	61.1
**Challenges**					
Excessive financial costs	990	38.5	28.3	27.5	28.6
Upsetting neighbours	528	19.5	15.6	14.1	15.2
Dog behavioural issues	1718	60.0	47.6	50.8	49.6
Increased responsibilities	2227	79.0	67.9	57.0	64.3
Dog training	2143	76.5	62.8	58.4	61.8
Compromised sleep quality	461	19.5	11.4	15.1	13.3

### Physical health

Participants expected an increase in walking (89%) to be the main physical health benefit of dog ownership, followed by an increase in physical fitness (52%), decrease in blood pressure (40%), and weight loss (30%) (Table 1). Table 2 presents the odds ratios describing the association between dog ownership status and the four most common expected physical, mental, and psychosocial health benefits. Compared with those who had never owned a dog before, after adjusting for age, sex, and education level, previous dog owners (OR 1.83; 95% CI: 1.17 to 2.88) had higher odds of expecting to walk more. Additionally, both previous (OR 1.37; 95% CI: 0.99 to1.89) and current (OR 1.62; 95% CI: 1.17 to 2.25) dog owners had higher odds of expecting a reduction in blood pressure as a result of dog ownership.

**Table 2:** Multiple logistic regression describing the associations between dog ownership status and the top four expected physical, mental and social health benefits from dog ownership.

		Model 1		Model 2	
Variable	Cases/n*	OR (95% CI)	P	OR (95% CI)	P
*Physical health*					
**Increase in walking**					
Never owned a dog	174/200	1	<0.001	1	<0.001
Previous dog owner	1742/1903	1.63 (1.04,2.53)	.031	1.83 (1.17,2.88)	.008
Current dog owner	1178/1362	0.99 (0.64,1.54)	.960	1.06 (0.67,1.65)	.815
**Increase in physical fitness**					
Never owned a dog	104/200	1	.007	1	.005
Previous dog owner	1026/1903	1.09 (0.81,1.46)	.567	1.15 (0.86,1.55)	.346
Current dog owner	655/1362	0.87 (0.65,1.17)	.357	0.91 (0.68,1.23)	.550
**Weight loss**					
Never owned a dog	63/200	1	<0.001	1	<0.001
Previous dog owner	620/1903	1.07 (0.78,1.46)	.684	1.08 (0.78,1.49)	.638
Current dog owner	347/1362	0.76 (0.55,1.05)	.096	0.77 (0.55,1.07)	.118
**Decrease in blood pressure**					
Never owned a dog	60/200	1	.001	1	.004
Previous dog owner	734/1903	1.49 (1.08,2.04)	.015	1.37 (0.99,1.89)	.054
Current dog owner	580/1362	1.76 (1.27,2.43)	.001	1.62 (1.17,2.25)	.004
*Mental health*					
**Increase in happiness**					
Never owned a dog	170/200	1	.009	1	.003
Previous dog owner	1685/1903	1.38 (0.92,2.09)	.124	1.67 (1.10,2.55)	.017
Current dog owner	1242/1362	1.82 (1.18,2.80)	.007	2.12 (1.36,3.29)	.001
**Decreased Stress**					
Never owned a dog	134/200	1	.012	1	.003
Previous dog owner	1400/1903	1.39 (1.02,1.90)	.038	1.57 (1.14,2.15)	.005
Current dog owner	1041/1362	1.60 (1.16,2.20)	.004	1.76 (1.27,2.44)	.001
**Reduced depression**					
Never owned a dog	105/200	1	.002	1	.001
Previous dog owner	1036/1903	1.08 (0.80,1.44)	.626	1.21 (0.90,1.63)	.207
Current dog owner	825/1362	1.37 (1.01,1.84)	.041	1.53 (1.13,2.08)	.006
**Decreased loneliness**					
Never owned a dog	110/200	1	.001	1	.001
Previous dog owner	1117/1903	1.18 (0.88,1.58)	.279	1.31 (0.97,1.77)	.081
Current dog owner	876/1362	1.49 (1.10,2.01)	.010	1.61 (1.19,2.20)	.002
*Psychosocial health*					
**Meet new friends**					
Never owned a dog	98/200	1	.855	1	.659
Previous dog owner	969/1903	1.08 (0.81,1.45)	.612	1.14 (0.85,1.54)	.378
Current dog owner	696/1362	1.09 (0.81,1.47)	.576	1.11 (0.82,1.50)	.501
**Meet new partner**					
Never owned a dog	25/200	1	.015	1	.115
Previous dog owner	131/1903	0.52 (0.33,0.82)	.005	0.62 (0.39,0.98)	.040
Current dog owner	95/1362	0.53 (0.33,0.84)	.007	0.63 (0.39,1.02)	.059
**Get to know neighbourhood**					
Never owned a dog	66/200	1	.333	1	.096
Previous dog owner	714/1903	1.22 (0.90,1.67)	.203	1.40 (1.02,1.91)	.037
Current dog owner	522/1362	1.27 (0.93,1.74)	.139	1.41 (1.03,1.94)	.034
**Companionship from dog**					
Never owned a dog	112/200	1	.327	1	.205
Previous dog owner	1170/1903	1.24 (0.93,1.67)	.147	1.31 (0.97,1.77)	.075
Current dog owner	835/1362	1.25 (0.93,1.69)	.077	1.29 (0.95,1.74)	.106

*Cases; number of participants that expect the benefit or challenge, n; number of participants in sample

Model 1 is unadjusted

Model 2 is adjusted for age, sex and education level

OR; Odds ratio, CI; Confidence interval

### Mental health

Most participants expected improvements across all mental health outcomes namely through increases in happiness (89%), and decreased stress (74%), loneliness (61%), and depression (57%) (Table 1). After adjusting for age, sex, and education level, current dog owners had higher odds of expecting increased happiness due to dog ownership (OR 2.12; 95% CI:1.36 to 3.29). Previous dog owners were also associated with increased odds, but to a lesser extent (OR 1.67; 95% CI: 1.10 to 2.55). A similar trend was observed for all remaining mental health outcomes; both current and previous dog owners had higher odds of expecting decreased stress, reduced depression, and decreased loneliness than participants who had never owned a dog.

### Psychosocial health

Over half of participants expected the dog to provide companionship (61%) and more opportunities to meet new friends (51%) (Table 1). Fewer participants expected they would get to know the neighbourhood (38%) or meet a new partner (7%). Both previous and current dog owners had higher odds of expecting they would get to know the neighbourhood (OR previous dog owners 1.40; 95% CI: 1.02 to 1.91; OR current dog owners 1.41; 95% CI: 1.03 to 1.94) and experience companionship from their dog (OR previous dog owners 1.31; 95% CI: 0.97 to 1.77; OR current dog owners 1.29; 95% CI 0.95 to 1.74) (Table 2). Previous and current dog owners had lower odds of expecting to meet a new partner (OR previous dog owners 0.62; 95% CI: 0.39 to 0.98; OR current dog owners 0.63; 95% CI: 0.39 to 1.02) compared with participants who had never owned a dog.

### Challenges

Among all respondents, increased responsibility (64%), dog training (62%), and dog behavioural issues (50%) were the most common expected challenges of dog ownership (Table 1). Less common expected challenges were excessive financial costs (28.6%), upsetting neighbours (15.2%) and compromised sleep quality (13.3%). Previous and current dog owners displayed an inverse association with all expected challenges compared to the reference group (Table 3). Being a current dog owner was associated with a 58% and 49% decrease in the relative odds of expecting increased responsibility (95% CI: 0.29 to 0.60) and dog training (95% CI: 0.36 to 0.73) respectively. Compared to never having owned a dog before, previous (OR 0.59, CI: 0.40 to 0.89) and current (OR 0.81, CI: 0.55 to 1.19) owners had lower odds of expecting dog ownership to compromise sleep quality.

**Table 3:** Multiple logistic regression describing the associations between dog ownership status and the expected challenges of dog ownership.

		Model 1		Model 2	
Variable	Cases/n*	OR (95% CI)	P	OR (95% CI)	P
**Excessive financial costs**					
Never owned a dog	77/200	1	.005	1	.051
Previous dog owner	538/1903	0.63 (0.46,0.85)	.002	0.72 (0.53,0.98)	.034
Current dog owner	375/1362	0.60 (0.44,0.82)	.001	0.68 (0.49,0.93)	.015
**Upsetting neighbours**					
Never owned a dog	39/200	1	.083	1	.190
Previous dog owner	297/1903	0.76 (0.53,1.11)	.156	0.85 (0.58,1.24)	.391
Current dog owner	192/1362	0.66 (0.45,0.97)	.036	0.74 (0.50,1.08)	.121
**Dog Behavioural issues**					
Never owned a dog	120/200	1	.002	1	.024
Previous dog owner	906/1903	0.60 (0.45,0.81)	.001	0.69 (0.51,0.93)	.015
Current dog owner	692/1362	0.68 (0.50,0.92)	.013	0.78 (0.57,1.05)	.105
**Increased responsibility**					
Never owned a dog	158/200	1	<0.001	1	<0.001
Previous dog owner	1292/1903	0.57 (0.40,0.81)	.002	0.68 (0.48,0.98)	.037
Current dog owner	777/1362	0.36 (0.25,0.51)	<0.001	0.42 (0.29,0.60)	<0.001
**Dog training and disciplining**					
Never owned a dog	153/200	1	<0.001	1	<0.001
Previous dog owner	1195/1903	0.53 (0.37,0.74)	<0.001	0.63 (0.45,0.89)	.008
Current dog owner	795/1362	0.44 (0.31,0.62)	<0.001	0.51 (0.36,0.73)	<0.001
**Compromised sleep quality**					
Never owned a dog	39/200	1	<0.001	1	.002
Previous dog owner	217/1903	0.53 (0.36,0.77)	.001	0.59 (0.40,0.87)	.007
Current dog owner	205/1362	0.73 (0.50,1.06)	.100	0.81 (0.55,1.19)	.272

*Cases; number of participants that expect the benefit or challenge, n; number of participants in sample

Model 1 is unadjusted

Model 2 is adjusted for age, sex and education level

OR; Odds ratio, CI; Confidence interval

## Discussion

To the authors’ knowledge, this is the largest study to investigate expectations of dog ownership among prospective dog owners. In accordance with previous studies [41], our findings indicate that most prospective adopters expect to receive a range of health benefits and some challenges. Dog ownership status appears to influence these expectations, with previous/current ownership history being associated with greater odds of expecting benefits and reduced odds of expecting challenges.

Prospective owners expected benefits across three primary areas of health, namely physical, mental and psychosocial health. The vast majority of participants expected increased physical activity levels (89%) and enhanced mental wellbeing, including increased happiness (89%) and decreased stress (74%). Just over half of participants expected companionship from the dog (61%) and decreased loneliness (61%). These results suggest that historic attempts such as “Walk the Dog” [45] intended to raise awareness of the potential health benefits of dog ownership may be effective. Several of the commonly expected benefits, such as increased physical activity [26] and decreased stress [36, 37] are, to some extent, supported by the literature investigating dog ownership and human health. Other common expectations, such as increased happiness or decreased loneliness are less supported by the current findings, with most published reports showing dog owners are no happier [46, 47] or less lonely than non-pet owners [48].

Our findings also demonstrate that previous ownership history and current status is associated with an increased likelihood of expecting benefits. Current and previous dog owners displayed consistently greater odds of expecting mental and psychosocial health benefits than non-owners, with one exception: meeting a new partner. The associations for anticipated physical health benefits show some variation with both previous and current dog owners displaying greater odds of expecting a decrease in blood pressure but only previous dog owners displaying greater odds of expecting an increase in walking. Perhaps current owners are already walking their dogs to the extent they see necessary, and do not expect a further increase in walking with the addition of another dog. Alternatively, current owners may anticipate that acquiring a second dog will reduce the need for them to walk their dogs, in the belief that play among dogs can reduce the need for owner-initiated exercise. It is plausible that participants with dog ownership history who are actively seeking another dog are more likely to expect physical, mental and psychosocial health benefits as they have possibly had positive experiences from previous ownership. It is also possible that current and previous dog owners exhibit some bias (selectively recalling positive experiences) when considering expectations for benefits, resulting from feelings of affection towards their current/previous dog(s). Supporting this interpretation, dog owners are known to exhibit a positive bias when viewing their own dog compared with the average dog [49]. It is also possible that drawing a sample from prospective owners who are willing to house a rescue dog inadvertently selects for those who have better dogmanship skills than the average owner and know how to cohabit and bond with dogs favourably [50].

Our study found the most frequently documented expected challenge was increased responsibility (64.3%) followed by dog training (61.8%) and dog behavioural issues (50%). These expectations broadly align with previous research that identified the primary concern of prospective adopters to be problem behaviour [40]. Our findings also mirror research investigating relinquishment, with the expectation of increased responsibility and dog training reflecting two of the most commonly cited relinquishment reasons; management problems and dog misbehaviour, respectively [51].

Respondents with current/previous ownership experience displayed lower odds than non-owners of expecting challenges, such as increased responsibility and dog training. This contradicts previous results that suggest greater animal-care knowledge is associated with an increased expectation for effort required in owning a dog [41]. These findings may be the result of positive biases exhibited by previous/current dog owners from previous ownership experience or even selection bias. A study of 307 unsuccessful dog adopters who returned their dog to an animal shelter, found previous dog ownership negatively influenced the likelihood of a successful adoption [51]. Specifically, current/previous dog-owners were more likely than first-time owners to return a dog to an animal shelter due to behavioural problems. This suggests they are less tolerant of misbehaviour [51] possibly due to unrealistic expectations from biases such as having grown accustomed to their previous dog’s needs and behavioural traits.

To optimise the human-dog bond, it is important that prospective dog owners’ expectations for benefits and challenges are realistic. This could be achieved through preadoption counselling, whereby veterinarians and shelter personnel educate future dog owners on the realities of dog ownership, based on current scientific evidence [3]. Staff should endeavour to educate all prospective owners of the potential for management challenges such as dog training difficulties, increased time demands and responsibility, regardless of their ownership history. Individuals adopting from animal shelters should also be informed of the limitations of behavioural testing in kennel environments [52] to ensure the adopters are aware of the possibility their dog may exhibit different behaviours in the home environment to the shelter.

This study has several limitations which must be acknowledged. Firstly, the cross-sectional study design limits our ability to determine a causal relationship between dog ownership status and expectations for benefits and challenges. Therefore, it is possible the observed associations are a result of reverse causation. For example, individuals with greater expectations for benefits and reduced expectations for challenges may be more interested in dog ownership initially, as reflected by their previous ownership history/current status. Secondly, residual confounding may have influenced our results, as there was no information collected on possible confounding factors such as socioeconomic status and race. A lack of information regarding the characteristics of non-respondents also precludes us from identifying differences between participants and non-respondents. Additionally, a convenience sampling method was used whereby individuals were invited to participate only if they were actively seeking a dog via the PetRescue website. In Australia, an estimated 16% of dogs are obtained through animal shelters [53]. Motivations for acquiring a rescue dog vary, but often include the belief that it is the ethically correct choice, the desire to save an animal from possible euthanasia or because it requires less financial outlay [3]. Given the difference in motivating factors, it is possible the demographics of our sample systematically differ from the general population of prospective dog owners. As mentioned above, this may be reflected in the current respondents’ dogmanship. Finally, the sample was predominantly female, middle aged and university educated which, to some extent, reflects the demographics of Australian dog owners who are most likely to be female and aged 18–49 [53]. Female respondents comprised a considerable proportion of the sample (85%), which parallels other studies in the field [41, 54]. Owner gender has been associated with differences in canine behaviour, although it is unknown whether these differences are the result of variations in perceptions of behaviour or true interactions between human sex and canine behaviour [55]. Nonetheless, the high percentage of female participants limits the generalizability of our findings to the wider population of prospective dog owners. The proportion of participants who had never owned a dog sample was also relatively small (6%).

In conclusion, our findings show that most prospective adopters expect to receive health benefits from dog ownership, such as increased walking, happiness and companionship and decreased stress and loneliness. Most prospective adopters also anticipate challenges, such as increased responsibility and training difficulties. Expectations vary depending on previous dog ownership experience, with current and previous dog owners generally displaying greater odds of expecting physical, mental and psychosocial health benefits and lower odds of expecting challenges than non-owners. Future longitudinal studies are required to further understand the differences in pre-adoption expectations and the health benefits and challenges resulting from dog ownership. Future research regarding the role of ownership history in prospective owners’ expectations could also benefit from the inclusion of previous/current dog owners who have no interest in acquiring a new dog. These findings could provide insight for the development of future interventions to optimise the human-dog bond and reduce relinquishment to animal shelters.

## Supporting information

S1 Table Survey questions relating to perceived mental, physical, and psychosocial health benefits and possible challenges associated with dog ownership. (DOCX)
