# Supplementary material for: Expectations for dog ownership: Perceived physical, mental and psychosocial health consequences among prospective adopters
Source: PLoS One. 2018 Jul 6;13(7):e0200276. doi: 10.1371/journal.pone.0200276 (PMC6034856; doi:10.1371/journal.pone.0200276)
Supplement: S1 Table — (DOCX) [file pone.0200276.s001.docx]

**S1 Table:** Survey questions relating to perceived mental, physical, and psychosocial health benefits and possible challenges associated with dog ownership

| **Question number** | **Question** | **Possible answers** |
| --- | --- | --- |
| 6 | Do you think that you will get any physical health benefits as a result of owning a dog? | Yes No |
| 7 | What physical health benefits do you think you will experience? | Increase in walking and other physical activity Increase in physical fitness Weight loss Decrease in blood pressure |
| 8 | Do you think that you will get any mental health benefits as a result of owning a dog? | Yes  No |
| 9 | What mental health benefits do you think you will experience? | Increase in happiness  Decreased stress levels  Reduced depression  Decreased loneliness |
| 10 | Do you think that you will get any benefits to your social life as a result of owning a dog? | Yes  No |
| 11 | What benefits to your social life do you think you will experience? | Opportunity to meet other dog owners to make friends  Opportunity to meet a new partner who is a dog owner  Opportunity to get to know your neighbourhood  Companionship from the dog |
| 12 | Do you think you will experience any challenges as a result of owning a dog? | Yes  No |
| 13 | What challenges do you think you might experience? | Excessive financial costs  Upsetting my neighbours  Behavioural issues with the dog  Increased responsibilities associated with caring for the dog  Training and disciplining the dog  Compromising my sleep quality |
